# The relationship between video display terminals (VDTs) usage and dermatologic manifestations : a cross sectional study

**DOI:** 10.1186/1471-5945-5-3

**Published:** 2005-04-03

**Authors:** Omid Aminian, Parvin Mansoori, Akbar Sharifian, Ehsan Rafeemanesh, Maria Mazaheri, Mohammadreza Iraniha

**Affiliations:** 1 Department of Occupational Medicine, Faculty of Medicine, Tehran University of Medical Sciences, Tehran, Iran

## Abstract

**Background:**

Recently, it has been observed that Video Display Terminals (VDTs) usage for long periods can cause some dermatological manifestations on the face. An analytical cross-sectional study was designed in order to determine this relationship.

**Methods:**

In this study, 600 office workers were chosen randomly from an organization in Tehran (Iran).

The subjects were then divided into two groups based on their exposure to VDTs.

306 workers were considered exposure negative (non VDT user) who worked less than 7 hours a week with VDTs. The remainders 294 were exposure-positive, who worked 7 hours or more with VDTs. The frequency of dermatologic manifestations was compared in these two groups.

**Results:**

In the exposure-positive and exposure-negative groups, the frequency of these dermatologic manifestations were 27 and 5 respectively.

After statistical analysis, a P.value of < 0.05 was obtained indicating a statistically significant difference between these two groups for dermatological manifestations.

**Conclusion:**

According to our study, there is a relationship between dermatologic manifestations on the face and exposure to VDTs.

## Background

There is growing evidence that long term exposure to the types of unfavorable working conditions that have been observed among some VDTs users might have serious health consequences [1].

Dermatological manifestations, especially on the face are one of these health outcomes [2].

The most common manifestations among these patients are nonspecific erythema, acne rosacea, seborrheic dermatitis, pruritus, burning sensation, and dry skin [3].

The prevalence of these manifestations among VDTs users ranges from 8–10% in a series of descriptive studies to 13.5% in other reports [4].

Radiation emissions from VDTs such as x-ray, ultraviolet, infrared are within acceptable levels and there is neither a connection between these radiations and health consequences, nor with any dermatological manifestations [4,5].

As a whole, the exact cause of these facial manifestations of VDTs users is not clear, but physical conditions of the workplace such as dryness, occupational stress [6], electrostatic fields [7], and to a lesser extent, electromagnetic fields of VDTs can play a role [8,9].

The relationship between working with VDTs and dermatological manifestations has not previously been investigated. Accordingly, this study was designed.

## Methods

An analytical cross-sectional study was conducted by using office workers of an organization in Tehran.

Age, gender and weekly hours of work with VDTs were considered as independent variables and dermatological manifestations on the face including erythema, acne rosacea, scaling, pruritus and burning sensation as dependent variables.

600 office workers were selected randomly from the workers with approximately same environmental conditions (temperature, humidity, light, etc.) and were divided into two groups according to VDT exposure in the past year.

The exposure-positive group consisted of those with 7 hours or more weekly exposure to VDTs in the workplace or home and the exposure-negative group with less than 7 hours weekly exposure.

The workers filled in a questionnaire and had a physical examination.

Non specific erythema, acne rosacea, scaling and seborrheic dermatitis were detected during examination and were considered as positive dermatologic findings. Pruritus and burning sensation which got worse or were produced by working with VDTs and alleviated after leaving the workplace were considered as acceptable positive dermatological findings.

## Results

251 workers were females and the remainders 349 were males.

Age and sex distribution of the workers is demonstrated in Table (1). The average age was 44.5 years.

**Table 1:** Frequency distribution of office workers with relation to age and gender.

**Age(year)**	**Male**	**Female**	**Sum**
25–29	15	10	25
30–34	48	41	89
35–39	64	57	121
40–44	95	56	151
45–49	99	66	165
50–54	25	20	45
55–59	3	1	4
Sum	349	251	600

294 workers had seven or more weekly hours of exposure and 306 had less than seven hours of weekly exposure in the past year.

In the exposure-positive group and exposure-negative groups 128 and 123 workers were females and 165 and 183 workers were males respectively.

The average weekly exposure in the exposure- positive group was 14 hours.

In the exposure-positive group 27 workers (16 female and 11 male) and in the exposure- negative groups 5 (3 female and 2 male) had dermatological manifestations respectively, as depicted in Table (2).

**Table 2:** Frequency of dermatologic manifestations in workers with more than 7 hours weekly exposure and less than 7 hours weekly exposure

**Dermatologic manifestation**	**Frequency in exposure negative group**	**Frequency in exposure positive group**
Nonspecific erythema	1	5
Rosacea acnea	1	2
Erythema,,Scaling	1	3
Itching, Burning	2	17
Sum	5	27

Frequency of the exposure-positive workers according to their weekly hours of exposure and the frequency of dermatologic manifestations is shown in Table (3).

**Table 3:** Frequency distribution of exposure positive group with weekly work hours and dermatologic manifestation

**Exposure time per week**	**People with dermatologic manifestations**	**Total people with exposure**	**Percent %**
7–11	4	104	3.85
12–16	11	126	8.6
17–21	4	32	12.5
22–26	6	24	25
27–31	2	8	25

(Chi-square, linear by linear association: statistic = 12.735, df = 1, p-value < 0.005)

## Discussion

Statistical analysis of the confounding factors (age and sex) was performed between the two groups and no statistically significant difference was observed.

Use of chi-square test led to a P.value of less than 0.05, indicating statistical differences between exposure-positive and exposure-negative groups for the above mentioned dermatological manifestations.

On the other hand, Table (3) clearly indicates that the frequency of dermatological manifestations of the face tend to increase with increasing weekly hours of work with VDTs (chi-square, linear by linear association: statistic = 12.735, df = 1, P.value < 0.005).

In a study conducted by Stenberg B. et al., psychosocial conditions and exposure to electromagnetic fields or conditions associated with such factors were related to an increased occurrence of skin symptoms.

The results also indicated that personal factors such as atopic dermatitis and physical exposure factors influencing indoor air quality, such as paper exposure and cleaning frequency were related to an increased prevalence of symptoms. The results suggest that skin symptoms reported by VDTs users have a multi-factorial causation [2]. According to other reports, mainly from Norway and Sweden, video display terminal work is suspected of causing skin rashes.

Three different studies, have tried to elucidate the question, and the results point to a possible relationship between VDT work and aggravation of some common skin diseases such as rosacea, seborrheic and atopic dermatitis, and acne. Whether this depends on physical, chemical, or psychological factors is still unknown [10].

## Conclusion

According to other studies and our own study, we can propose a relationship between dermatological manifestations on the face and exposure to VDTs and the probability of the occurrence of these manifestations increase with increasing exposure time.

Based on our findings and those of others, we recommend that in workers with long time exposures to VDTs who display dermatological manifestations of the face, occupational history of working with VDTs, weekly hours of exposure, and effects of exposure on their symptoms should be considered.

## Competing interests

The author(s) declare that they have no competing interests.

## Authors' contributions

AO participated in the design of the study and performed the data collection.

MP conceived of the study, and participated in its design and coordination.

SA drafted the manuscript and coordination.

RE performed the statistical analysis.

IM and MM drafted the manuscript.

All authors read and approved the final manuscript.

## Pre-publication history

The pre-publication history for this paper can be accessed here:


